# LLM-enabled Natural History Study Analysis to Support Rare Disease Research

**DOI:** 10.64898/2026.09.17.26363303

**Published:** 2026-09-20

**Authors:** Kevin Li, Eric Sid, Qian Zhu

## Abstract

Background: Rare diseases affect an estimated 300 million people worldwide, yet the research needed to guide diagnosis and treatment is often fragmented across multiple unstructured literature sources. Natural history studies (NHS) are a key source of this evidence, but manually extracting structured information from NHS publications can be tedious and does not scale. Methods: We have developed a proof-of-concept for an information extraction pipeline testing three opensource large-language models (LLMs) -- Athena-v3-AWQ, Google's Gemma3-27B, and Meta's Llama-3.1-70B-Instruct, to extract key NHS characteristics from PubMed abstracts curated from a Chan Zuckerberg Initiative disease research state model corpus (302 gold-standard and 8,338 full-corpus abstracts), and compared the models on efficiency, extraction completeness, and expert-rated accuracy. Results: All three models processed abstracts with success rates exceeding 99%. However, Gemma achieved the best overall performance, with the highest expert-rated accuracy (68.0% of outputs rated "good" vs. 36.0% for Llama and 10.0% for Athena) and the fastest runtime on the full corpus (~16 minutes for 3,547 abstracts), despite Llama scoring higher on the automated Token F1 metric (0.874 vs. 0.723), highlighting a divergence between automated and human evaluation. Athena's lower performance was largely attributable to verbatim copying rather than synthesis of extracted content. Conclusions: These findings illustrate how locally deployed open-source LLMs can extract structured NHS characteristics at scale, thus supporting their use to accelerate evidence synthesis in rare disease research.

## Full Text Availability

The license terms selected by the author(s) for this preprint version do not permit archiving in PMC. The full text is available from the preprint server.

